# Effects of prolonged methylphenidate treatment on amygdala reactivity and connectivity: a randomized controlled trial in stimulant treatment-naive, male participants with ADHD

**DOI:** 10.1093/psyrad/kkab013

**Published:** 2021-10-22

**Authors:** Antonia Kaiser, Marco A Bottelier, Michiel B de Ruiter, Michelle M Solleveld, Hyke G H Tamminga, Cheima Bouziane, Hilde M Geurts, Ramon J L Lindauer, J J Sandra Kooij, Paul J Lucassen, Anouk Schrantee, Liesbeth Reneman

**Affiliations:** Amsterdam UMC, University of Amsterdam, Department of Radiology and Nuclear Medicine, Amsterdam Neuroscience, Amsterdam, 1105 AZ, the Netherlands; Amsterdam UMC, University of Amsterdam, Department of Radiology and Nuclear Medicine, Amsterdam Neuroscience, Amsterdam, 1105 AZ, the Netherlands; University Medical Center Groningen, Child Study Center, Accare, Groningen, 9713GZ, the Netherlands; Amsterdam UMC, University of Amsterdam, Department of Radiology and Nuclear Medicine, Amsterdam Neuroscience, Amsterdam, 1105 AZ, the Netherlands; Netherlands Cancer Institute, Division of Psychosocial Research and Epidemiology, Amsterdam, 1066CX, the Netherlands; Amsterdam UMC, University of Amsterdam, Department of Radiology and Nuclear Medicine, Amsterdam Neuroscience, Amsterdam, 1105 AZ, the Netherlands; Amsterdam UMC, University of Amsterdam, Department of Radiology and Nuclear Medicine, Amsterdam Neuroscience, Amsterdam, 1105 AZ, the Netherlands; University of Amsterdam, Dutch Autism & ADHD Research Center, Department of Psychology, Amsterdam, 1018WT, the Netherlands; Amsterdam UMC, University of Amsterdam, Department of Radiology and Nuclear Medicine, Amsterdam Neuroscience, Amsterdam, 1105 AZ, the Netherlands; University of Amsterdam, Dutch Autism & ADHD Research Center, Department of Psychology, Amsterdam, 1018WT, the Netherlands; Amsterdam UMC, University of Amsterdam, Department of Child and Adolescent Psychiatry, Amsterdam, 1105AZ, the Netherlands; Academic Centre for Child and Adolescent Psychiatry, Levvel, Amsterdam, 1076EC, the Netherlands; Expertise Center Adult ADHD, PsyQ, The Hague, 2512VA, the Netherlands; Amsterdam UMC, Vrije Universiteit, Amsterdam Public Health Research Institute, Department of Psychiatry, Amsterdam, 1105AZ, the Netherlands; University of Amsterdam, Brain Plasticity Group, Swammerdam Institute for Life Sciences, Amsterdam, 1012WX, The Netherlands; Amsterdam UMC, University of Amsterdam, Department of Radiology and Nuclear Medicine, Amsterdam Neuroscience, Amsterdam, 1105 AZ, the Netherlands; Amsterdam UMC, University of Amsterdam, Department of Radiology and Nuclear Medicine, Amsterdam Neuroscience, Amsterdam, 1105 AZ, the Netherlands

**Keywords:** ADHD, fMRI, emotional dysregulation, internalizing symptoms, amygdala, face-matching paradigm

## Abstract

**Background:**

Problems with emotional processing are widely reported in individuals with attention-deficit/hyperactivity disorder (ADHD). Although methylphenidate (MPH) effectively alleviates inattention and hyperactivity symptoms in ADHD, its effects on emotional processing and internalizing symptoms have remained elusive. While we previously found that acute MPH administration modulated neural mechanisms underlying emotional processing in an age-dependent manner, the effects of prolonged administration remained unknown.

**Objectives:**

Therefore, we investigated: (i) whether prolonged MPH treatment influences neural substrates (amygdala reactivity and connectivity) of emotional processing, and (ii) whether these effects are modulated by age.

**Methods:**

The “effects of Psychotropic drugs On Developing brain-MPH” (“ePOD-MPH”) randomized controlled trial was a 16-week double-blind, placebo-controlled, multi-center trial with MPH in 50 boys (10–12 years of age) and 49 men (23–40 years of age), all stimulant treatment-naive and diagnosed with ADHD. Participants performed an emotional face-matching task during functional magnetic resonance imaging. We assessed their symptoms of ADHD and internalizing symptoms at baseline, during the trial (8 weeks), and 1 week after the trial end (17 weeks).

**Results and Conclusions:**

We did not find effects of prolonged MPH treatment on emotional processing, as measured by amygdala reactivity and connectivity and internalizing symptoms in this trial with stimulant treatment-naive participants. This differs from our findings on emotional processing following acute MPH administration and the effects of prolonged MPH treatment on the dopamine system, which were both modulated by age. Interestingly, prolonged MPH treatment did improve ADHD symptoms, although depressive and anxiety symptoms showed a medication-independent decrease. Furthermore, our data indicate that baseline internalizing symptoms may be used to predict MPH treatment effects on ADHD symptoms, particularly in (male) adults with ADHD.

## Introduction

Methylphenidate (MPH), the primary pharmacological treatment for attention-deficit/hyperactivity disorder (ADHD), effectively alleviates symptoms of inattention and hyperactivity in individuals with ADHD. However, individuals with ADHD also present difficulties in emotion processing, independent of other comorbidities (Lenzi *et al*., 2018). Divergent emotional processing in ADHD has been linked to both externalizing symptoms, such as conduct problems (Gillberg *et al*., 2004), and to internalizing symptoms associated with symptoms of anxiety and depression (Jarrett and Ollendick, 2008; Sciberras *et al*., 2014). Additionally, it has been found to impact the quality of life of individuals with ADHD seriously and was in fact associated with poorer daily life functioning (Kuhne *et al*., 1997; Riley *et al*., 2006; Sciberras *et al*., 2014; Schei *et al*., 2016). Clinical experience suggests that MPH may positively affect emotion regulation, as supported by a recent meta-analysis (Lenzi *et al*., 2018).

One possible pathophysiological substrate underlying emotional processing in ADHD may involve a dysfunctional striato-amygdalo-medial prefrontal cortical network (Shaw *et al*., 2014). Likewise, in ADHD, specific brain regions related to emotion processing have shown altered connectivity to the rest of the brain (Icer *et al*., 2018). For example, more emotional problems, particularly externalizing symptoms, were associated with a hyperconnectivity of the cortico-amygdalar network, including the anterior cingulate cortex, both in children and adolescents (Hulvershorn *et al*., 2014; Hafeman *et al*., 2017; Damiani *et al*., 2020). Furthermore, different self-regulation problem dimensions were associated with stronger negative whole-brain functional connectivity patterns in children (Rohr *et al*., 2020). Additionally, in adolescents with ADHD (aged 11–16 years), hyperreactivity and -connectivity of the amygdala were reported in response to fearful faces, which was notably increased further after MPH abstinence (Posner *et al*., 2011b). Also, an acute MPH challenge has been found to normalize altered resting-state circuits in children and adults with ADHD (Pereira-Sanchez *et al*., 2021). However, even though a few studies have shown these positive effects of stimulants on internalizing emotional symptoms (Biederman *et al*., 2009; Coughlin *et al*., 2015), the exact neural mechanisms underlying changes in emotional processing in ADHD remained unclear, especially following more prolonged durations of stimulant treatment.

Increasing preclinical evidence suggests that the effects of ADHD medication are modulated by age (Andersen, 2003, 2005; Urban *et al*., 2012), which we also found to be the case in a clinical trial comparing boys and adults with ADHD (Schrantee *et al*., 2016; Solleveld *et al*., 2017). Accordingly, we have previously shown that acute MPH administration modulates one of the functional neural mechanisms underlying emotional processing, i.e. amygdala reactivity, in an age-dependent manner (Bottelier *et al*., 2017). Additionally, preclinical studies have shown that prolonged treatment during adolescence induced anxiety and depressive-like behavior (Bolaños *et al*., 2003) and increased impulsivity during adulthood (Somkuwar *et al*., 2016). The most comprehensive study on long-term effects of ADHD medication to date, i.e. the multi-modal treatment study of ADHD (MTA), found that children treated with ADHD medication had higher rates of anxiety and depression (19.1%) than children receiving behavioral therapy only (4.3%), as measured 6 years after treatment onset. However, this effect had disappeared after 8 years (Molina *et al*., 2009).

Therefore, in the current study, we set out to: (i) investigate whether prolonged treatment with MPH influences internalizing symptoms and the neural substrates underlying emotional processing in stimulant-naive participants with ADHD, and (ii) assess whether these effects are modulated by age. Based on the literature, we expected that MPH would increase amygdala reactivity and the connectivity to the prefrontal cortex during an emotional face-matching, functional magnetic resonance imaging (fMRI) paradigm in children but not, or less so, in adults.

## Methods

The present study is part of the “effects of Psychotropic drugs On Developing brain-MPH” (“ePOD-MPH”) randomized controlled trial (RCT), which was a 16-week double-blind, randomized, placebo-controlled, multi-center trial with MPH, and a blinded endpoint evaluation in stimulant treatment-naive participants with ADHD (Bottelier *et al*., 2014). The primary objective of the ePOD-MPH RCT was to report on the age-dependent effects of MPH on the outgrowth of the dopaminergic system, as published elsewhere (Schrantee *et al*., 2016). The current study investigated the secondary outcome measures, namely functional measures underlying these changes, including emotional processing. The study protocol applied the code of medical ethics and was registered by the Central Committee on Research Involving Human Subjects (an independent registry) on March 24, 2011 (identifier NL34509.000.10) and subsequently at the Netherlands National Trial Register (identifier NL2955/NTR3103). The enrollment started with the first patient on October 13, 2011, ended on June 15, 2015, and was monitored by the Clinical Research Unit of the Amsterdam University Medical Center, University of Amsterdam, Amsterdam, the Netherlands.

### Participants

We included 50 stimulant treatment-naive boys (10–12 years of age) and 49 stimulant-treatment naive men (23–40 years of age) in the ePOD-MPH RCT. They were diagnosed with ADHD and recruited through clinical programs at the Child and Adolescent Psychiatry Center Triversum (Alkmaar), the Department of Child and Adolescent Psychiatry at the Bascule/AMC (Amsterdam), and the PsyQ Mental Health Facility (The Hague).

An experienced psychiatrist (MAB) diagnosed all children and adults. They met criteria for ADHD according to the Diagnostic and Statistical Manual of Mental Disorders (DSM-IV, 4th edition), as confirmed by a structured interview, i.e., the Diagnostic Interview Schedule for Children [NIMH-DISC-IV: authorized Dutch translation (Ferdinand and van der Ende, 1998)] and the Diagnostic Interview for ADHD (DIVA 2.0) for adults (Kooij, 2012). The DSM-IV requirement of at least six inattention or hyperactivity/impulsivity symptoms was applied to both children and adults. Exclusion criteria were: comorbid axis I psychiatric disorders requiring treatment with medication at study entry; and a history of major neurological or medical illness or clinical treatment with drugs influencing the dopaminergic system (for adults before 23 years of age), such as stimulants, neuroleptics, antipsychotics, and/or D_2/3_ agonists. More detailed inclusion and exclusion criteria are listed in the Supplementary Methods. All participants and parents or legal representatives of the children provided written informed consent after receiving a complete description of the study.

### Intervention, randomization, and blinding

**Figure 1:**
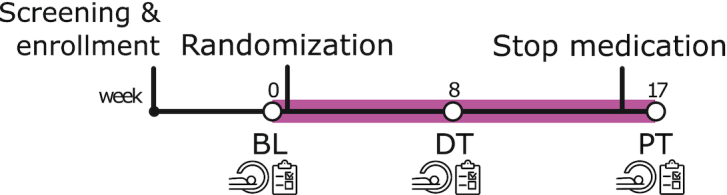
Timeline of the ePOD-MPH RCT. We measured fMRI activity and connectivity on an emotional face-matching task at three time points: at BL before randomization, 8 weeks during treatment (DT), and 1 week after the trial end (PT). Furthermore, we assessed clinical measures of ADHD, anxiety, and depression at these same timepoints.

After baseline (BL) assessments, we stratified participants by age and randomized them to either placebo or MPH treatment (1:1), using a permuted block randomization scheme generated by the local Clinical Research Unit. The treating physician prescribed the study medication under double-blind clinical guidance (reduction of ADHD symptoms) following Dutch treatment guidelines. Participants received oral dosages of short-acting MPH starting with 1–2 doses of 0.3 mg/kg daily. Dosages were increased weekly with 5–10 mg/day to a maximum of 50 mg/day until the target clinical dosage was reached, in line with clinical guidelines in the Netherlands. If, after in- or decreasing the dosage, serious side-effects occurred, the participant returned to the previous dosage and dosage modifications were more gradual thereafter. Decisions about dosage modifications were always and only done by the treating psychiatrist (mean dosage per person in Supplementary Results). Participants, care providers, and research personnel were blinded to the treatment condition (Supplementary Methods for further details). The Medical Center Alkmaar hospital pharmacy assigned participants to a specific allocation, using sequentially numbered containers. The appearance of the placebo tablet was identical to the MPH tablet and was manufactured and labeled according to GMP guidelines (2003/94/EG). We obtained data at three timepoints: at BL, at 8 weeks into treatment (during treatment = DT), and 1 week after the treatment had ended (posttreatment = PT) (Fig. 1). Short-acting MPH has a half life (*t*_1/2_) of approximately 2 hours, therefore, MPH is cleared approximately 10 hours after the last MPH administration. We used a wash-out period of 1 week to ensure that no acute effects of MPH influenced the PT assessment. Adherence to the study medication was monitored at each of the control visits and was expressed as a percentage, based on the number of tablets remaining divided by the number of tablets that should have remained (based on the daily dose, adjusted at each of the control visits). Adult participants received coaching sessions, and parents of children received psychoeducation.

### Clinical and behavioral variables

In children, we assessed ADHD severity, anxiety, and depressive symptoms using the disruptive behavior disorder rating scale (DBD-RS) (Pelham Jr *et al*., 1992), Child Depression Inventory (CDI) (Kovacs, 1985), and the child version of the Screen for Child Anxiety Related Disorders (SCARED) (Muris *et al*., 2007). In adults, we used the Attention Deficit Hyperactivity Disorder-Self Report (ADHD-SR) (Kooij, 2012), Beck's Depression Inventory (BDI) (Beck *et al*., 1961), and Beck's Anxiety Inventory (BAI) (Beck *et al*., 1988). We assessed all clinical scales at BL, DT, and PT (Fig. 1). Behavioral response data (accuracy and reaction times) of the fMRI task were extracted from E-Prime.

### fMRI

Participants performed an emotional face-matching fMRI paradigm at BL, DT, and PT (Fig. 1). We presented a practice run before the first MRI scan, and used two versions of the task to minimize learning effects. The emotional face-matching paradigm consisted of a blocked design and was adapted from a task previously used to assess drug effects on amygdala reactivity (Hariri *et al*., 2002; Bottelier *et al*., 2017). The emotional stimuli consisted of angry and fearful faces, and the neutral stimuli consisted of ellipses assembled from scrambled faces (Supplementary Fig. S1). During the task, we recorded reaction-time to button press and accuracy.

The MRI study was performed on a 3T Philips scanner (Philips Healthcare, Best, Netherlands) using an eight-channel receive-only head coil. Eight children and one adult were scanned on a 3T Phillips scanner at a different center (Philips Healthcare, Best, Netherlands). A 3D T1-weighted anatomical scan was acquired for registration purposes, and fMRI data were acquired using a single-shot echo-planar imaging sequence (parameters: TR/TE = 2300/30 ms, resolution = 2.3 × 2.3 × 3 mm, 39 sequential slices, FA = 80°, dynamics = 70). Preprocessing was performed using FMRIPREP v.1.2.3 (Esteban *et al*., 2019, 2020) (RRID: SCR_016 216). Each T1-weighted (T1w) scan was normalized to MNI space. Functional data preprocessing included motion correction (FLIRT), distortion correction (3dQwarp), followed by coregistration to the T1w. Independent component analysis-based Automatic Removal Of Motion Artifacts (ICA-AROMA) was used to generate nonaggressively denoised data. Subsequently, data were spatially smoothed (6mm FWHM) and high pass-filtered (100s) within FSL/FEAT (FSL/FEAT v.6.00; RRID: SCR_002 823) (Supplementary Methods for further detail).

FMRI data were entered into the first-level analysis in FSL/FEAT (Jenkinson *et al*., 2012). For our regions of interest (ROI) analyses of the emotional face-matching task, mean signal intensity for the left and right amygdala (Posner *et al*., 2011b) was extracted from the first level contrasts using masks from the Harvard–Oxford atlas (thresholded at 50%). To explore whole-brain activity in the main task contrasts (faces vs shapes; shapes vs faces), the first-level contrast-of-parameter-estimates (COPE) maps were analyzed pairwise using nonparametric permutation testing (5000 permutations) in FSL Randomise. Thresholds for all analyses were initially set at *P* *<* 0.05 with family-wise error corrections using threshold-free cluster enhancement (Winkler *et al*., 2014). From a total of 198 MRI scans, 34 scans could not be entered into the statistical analysis (17.2%). Exclusion criteria for MRI scans were: technical problems (0.6%), mean frame-wise displacement >0.5 mm (2%), scrubbing >15% (1.5%), drop-out (8.6%), or incomplete understanding of the task <70% accuracy (4.5%).

Psychophysiological interaction (PPI) analyses were conducted to assess connectivity during the emotional face-matching task. The left and right amygdala were chosen as seed regions and separately entered into two first-level models. Whole-brain analyses were performed as described before, using the first level data of the PPI analysis.

### Statistical analysis

All statistical analyses were conducted using R v.3.5.3 (R Development Core Team, 2011). Clinical and behavioral variables were analyzed intention-to-treat, and fMRI activity was analyzed per protocol with the significance level set at *P* *<* 0.05 (two-sided). All data were checked for normality and, in the case of nonnormality, transformed accordingly. To account for missing data points and the longitudinal nature of the RCT, linear mixed-effects models were used to analyze clinical and behavioral variables and fMRI activity to investigate the main effect of scan session (BL, DT, PT), medication group (placebo, MPH), and age group (children, adults), and its corresponding interaction effects using the lme4 package (Bates *et al*., 2014). For the amygdala reactivity data, the average framewise displacement per participant was added to the model as a covariate. A variable representing the scanner that was used was tested as a possible covariate and found to not contribute significantly. Additionally, we tested whether leaving out the participants with comorbidities changed the results of our analyses. Model selection was based on an adjusted top-down procedure, in which the resulting models were compared using the Bayesian information criterion (BIC), and consequently, the model best capturing the data was reported using approximate *F*-tests based on the Kenward–Roger approach (Kenward and Roger, 1997). Follow-up pairwise comparisons were corrected for multiple testing using a Sidak correction. Exploratory prediction analyses were done using linear models (lm); BL ADHD severity scores were included as a covariate.

## Results

### Clinical characteristics and randomization

A total of 99 participants with ADHD were randomized to either MPH or placebo. No serious adverse events were reported in any of the participants. Treatment groups did not differ in age, intelligence quotient (IQ), depressive or anxiety symptoms, and ADHD severity at BL (Table 1). One adult in the placebo group had a current panic disorder. Discarding the data from this participant did not change the results, and therefore we decided to include these data in the analyses.

**Table 1:** Characteristics of all participants enrolled in the RCT.

	Children			Adults		
	MPH	Placebo	Statistics	MPH	Placebo	Statistics
	*n* = 25	*n* = 25		*n* = 24	*n* = 24	
	mean ± SD	mean ± SD		mean ± SD	mean ± SD	
Age (y)	11.4 ± 0.8	11.3 ± 0.9	*t*(48) = −0.42, *P =* 0.67	28.6 ± 4.6	29.0 ± 4.9	*t*(46) = −0.59, *P =* 0.55
Estimated IQ^1^	104.8 ± 21.0	103.4 ± 15.1	*t*(46) = 0.28, *P =* 0.77	107.9 ± 8.8	107.9 ± 6.4	*t*(42) < 0.01, *P* > 0.9
**ADHD subtype, no**.						
Inattentive	14	14		11	5	
Hyperactive/impulsive	0	1		0	0	
Combined	11	10		13	19	
**ADHD symptoms**						
DBD-RS^2^ inattention	21.7 ± 3.2	22.8 ± 3.4	*t*(47) = −1.18, *P =* 0.24	–	–	
DBD-RS^2^ hyperactivity	15.0 ± 5.0	16.4 ± 6.3	*t*(47) = −0.90, *P =* 0.37	–	–	
ADHD-SR^3^	–	–		31.8 ± 9.9	31.1 ± 9.7	*t*(46) = 0.24, *P =* 0.82
**Comorbid psychiatric disorders:**						
Panic disorder	0	0		0	1	
Depressive symptoms^4^	8.1 ± 4.4	8.5 ± 4.6	*t*(46) = 0.87, *P =* 0.79	6.3 ± 5.5	8.1 ± 6.2	*t*(44) = *−*1.05, *P =* 0.30
Anxiety symptoms^4^	25.9 ± 17.4	29 ± 16.9	*t*(47) = −0.63, *P =* 0.53	9.1 ± 6.7	8.9 ± 8.2	*t*(46) = 0.08, *P =* 0.94
Adherence (%)	84 ± 15	80 ± 18	*t*(44) = 0.88, *P =* 0.38	90 ± 8	86 ± 8	*t*(43) = 1.98, *P =* 0.06
Average MPH dose (mg)	31.3 ± 7.3	34.4 ± 7.9	*t*(45) = 0.80, *P =* 0.43	51.1 ± 9.8	55.2 ± 8.7	*t*(42) = −0.18, *P =* 0.86

1 For children: Wechsler Intelligence Scale for Children (WISC) (Kort *et al*., 2002). For adults: National Adults Reading Test (NART) (Schmand *et al*., 1992); ^2^DBD-RS (Pelham Jr *et al*., 1992); and ^3^ADHD-SR (Kooij, 2012).

4 Depressive symptoms and anxiety symptoms: children, CDI (Kovacs, 1985) and SCARED (Muris *et al*., 1998); adults, BDI (Beck *et al*., 1961) and BAI (Beck *et al*., 1988).

### Treatment assignment

In Supplementary Fig. S2, treatment allocation and drop-out rates are reported according to CONSORT standards. One adult was excluded from the analysis due to undisclosed previous stimulant treatment. Eight adults underwent the PT scan at 8 weeks instead of at 17 weeks of the trial due to significant technical changes (software upgrade) to the MRI scanner. The mean treatment duration did not differ between both treatment groups in adults (*t*(42) = −0.02, *P =* 0.98) or children (*t*(45) = 0.15, *P =* 0.88). Medication conditions did not differ in age, IQ, or ADHD, depression and anxiety symptoms, or motion parameters after exclusion of scans (Supplementary Table S1).

### Behavioral outcomes

**Figure 2:**
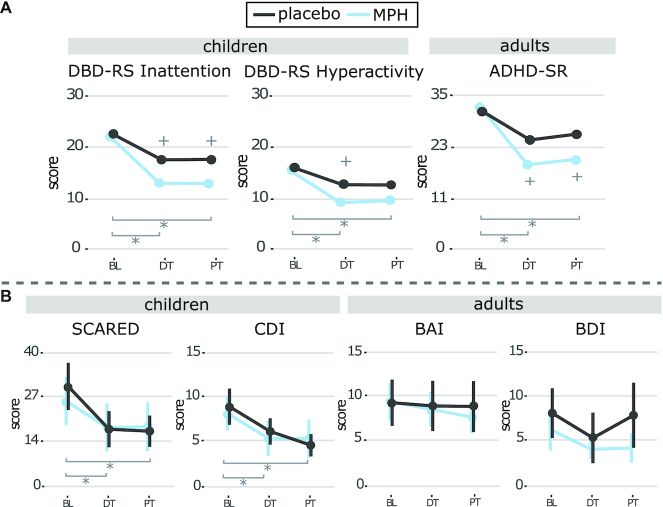
(A) ADHD symptoms. Line graphs show mean with 95% CI ADHD symptoms. A medication (MPH, placebo) × session (BL, DT, PT) effect was found for ADHD symptoms in adults and the inattention subscale in children but not the hyperactivity subscale. Post hoc tests revealed that ADHD symptoms in the MPH group decreased significantly more than in the placebo group from BL to DT and continued to be lower at PT in adults and the inattention subscale in children. We found a significantly larger decrease for the MPH group than placebo at DT for the hyperactivity subscale in children, but not at PT. (B) Clinical variables. Line graphs show the mean with 95% CI of anxiety, and depression scores. A main effect of session (BL, DT, PT) was found for anxiety and depressive symptoms in children, and depressive symptoms in adults. For depression and anxiety symptoms, both the MPH and placebo conditions in children showed improvements from BL to PT, but not adults. * = post hoc effect of session; + = post hoc effect of medication condition.

Linear mixed-effects model analyses showed a significant medication × scan-session effect for ADHD symptoms in adults (*F*(2,78) = 4.82, *P =* 0.01). Post hoc tests revealed that ADHD symptoms in the MPH group decreased significantly more than in the placebo group from BL to 8 weeks during treatment (DT) and continued to be lower 1-week PT (ADHD-SR: DT: *t*(101) = −2.21, *P =* 0.03; PT: *t*(95) = −2.33, *P =* 0.02). In children, the inattention subscale showed a significant medication × scan-session effect (DBD-RS-A: *F*(2,83) = 5.47, *P* *<* 0.01), but not the hyperactivity subscale (DBD-RS-H: *F*(3,90) = 2.48, *P =* 0.07). Post hoc tests revealed that inattention symptoms in the MPH group decreased significantly more than in the placebo group from BL to 8 weeks DT and continued to be lower 1 week PT (DBD-RS-A: DT: *t*(116) = −3.62, *P* *<* 0.01; PT: *t*(111) = −3.77, *P* *<* 0.01). For the hyperactivity subscale in children, we found a significantly larger decrease for the MPH group than for placebo at DT, but not at PT (DBD-RS-H: DT: *t*(94) = −2.16, *P =* 0.03; PT: *t*(88) = −1.87, *P =* 0.07) (Fig. 2A; Supplementary Table S2).

For anxiety symptoms, a main effect of scan session was only found in the children (SCARED: *F*(2,93) = 22.70, *P* *<* 0.01; BAI: *F*(2,85) = 2.01, *P =* 0.14); both the MPH and placebo conditions in children showed improvement from BL to 1 week PT (MPH: *t*(96) = 3.32, *P* *<* 0.01; placebo: *t*(94) = 5.17, *P* *<* 0.001) (Fig. 2B; Supplementary Table S2).

For depressive symptoms, in both children and adults, a main effect of scan session was found (CDI: *F*(2,91) = 38.17, *P* *<* 0.01; BDI: *F*(2,44) = 4.05, *P =* 0.02). Post hoc tests showed that both the MPH and placebo conditions in children improved from BL to 1 week PT (CDI: MPH: *t*(93) = 5.36, *P* *<* 0.001; placebo: *t*(92) = 6.58, *P* *<* 0.001); however, in adults, the effect was driven by a small effect of the placebo group from BL to DT (BDI: *t*(90) = 2.77, *P =* 0.02). No treatment effects were found from BL to PT (BDI: MPH: *t*(91) = 1.45, *P =* 0.32; placebo: *t*(90) = 0.79, *P =* 0.71) (Fig. 2B; Supplementary Table S2).

**Figure 3:**
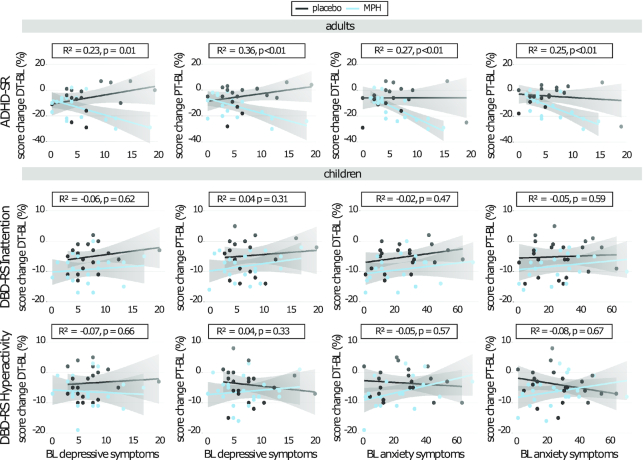
Prediction analysis. Scatterplots showing linear regressions between BL anxiety or depression symptoms and ADHD symptom severity change from BL to DT and BL to PT. Higher clinical scores at BL significantly predicted a higher decrease in ADHD symptom severity in adults treated with MPH (but not placebo) and not in children.

Prediction analysis revealed a significant interaction effect of BL depressive and anxiety symptoms and medication condition on ADHD symptom change from BL to DT and BL to PT in adults, but not children (DT-BL: BDI: *F*(4,31) = 8.93, *P* *<* 0.01; BAI: *F*(4,32) = 10.70, *P* *<* 0.01; PT-BL: BDI: *F*(4,34) = 13.26, *P* *<* 0.01; BAI: F(4,35) = 13.67, *P* *<* 0.01). Post hoc tests showed that adults in the placebo condition did not show any relation between the clinical variables and ADHD symptom change. In contrast, a negative association was found in the MPH condition, meaning higher BL depression and anxiety scores predicted a larger ADHD symptom severity decrease (Fig. 3).

### fMRI results

#### Emotional face-matching paradigm

As expected, the emotional face-matching task elicited activity in the bilateral amygdala, bilateral and medial prefrontal cortex, and bilateral occipital and parietal areas, including the fusiform gyrus at BL. For activation maps, see Bottelier *et al*. (2017).

**Figure 4:**
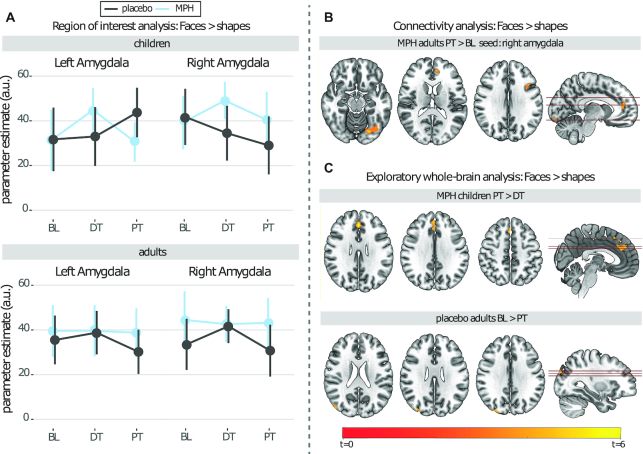
FMRI results of the emotional face-matching task. (A) ROI analysis. Line graphs display mean with 95% CI, showing no significant interaction or main effects of session (BL, DT, PT) and medication (MPH, placebo). (B) PPI-analysis. Whole-brain maps per group showed increased connectivity between the right amygdala and the occipital fusiform gyrus, paracingulate gyrus, and inferior frontal gyrus in adults treated with MPH from BL to PT. (C) Exploratory whole-brain analysis. Whole-brain maps show increased reactivity in the superior frontal cyrus and paracingulate cortex in children treated with MPH from BL to DT and decreased reactivity in the lateral occipital cortex in adults treated with placebo from BL to PT.

Linear mixed-effects model analyses did not show a significant age × medication × scan-session interaction on left or right amygdala reactivity (left: *F*(11,181) = 0.91, *P =* 0.53; right: *F*(10,172) = 0.74, *P =* 0.69), nor a significant scan-session × medication interaction in children (left: *F*(5,70) = 1.23, *P =* 0.30; right: *F*(5,69) = 1.22, *P =* 0.31) or adults (left: *F*(5,93) = 0.69, *P =* 0.63; right: *F*(5,93) = 1.17, *P =* 0.33), nor any main effects of scan session (children: left: *F*(2,63.61) = 0.68, *P =* 0.51; right: *F*(2,60.58) = 1.27, *P =* 0.29; adults: left: *F*(2,83.85) = 0.86, *P =* 0.43; right: *F*(2,82.64) = 0.85, *P =* 0.43), or medication (children: left: *F*(1,39.45) = 0.14, *P =* 0.70; right: *F*(1,40.54) = 0.85, *P =* 0.36; adults: left: *F*(1,42.50) = 1.12, *P =* 0.30; right: *F*(1,43.12) = 2.57, *P =* 0.12) (Fig. 4A; Supplementary Table S2). Furthermore, none of the clinical questionnaires of ADHD, depression, or anxiety in either the children or adults correlated with the left or right amygdala reactivity in any of the sessions (Supplementary Table S4).

Accuracy and reaction time (RT) data did not show any medication × scan-session interaction (children: accuracy faces: *F*(5,72.66) = 0.99, *P =* 0.50; accuracy shapes: *F*(3,68.83) = 1.24, *P =* 0.30; RT faces: *F*(5,66.93) = 1.06, *P =* 0.39; RT shapes: *F*(5,87.10) = 0.84, *P =* 0.52; adults: accuracy faces: *F*(5,85.32) = 0.09, *P =* 0.66; accuracy shapes: *F*(3,82.97) = 0.04, *P =* 0.99; RT faces: *F*(3,81.50) = 1.36, *P =* 0.26; RT shapes: *F*(5,67.13) = 1.02, *P =* 0.42), nor medication main effects (Supplementary Table S2, Supplementary Fig. S4).

#### Connectivity and exploratory whole-brain analyses

PPI analysis per group indicated that MPH increased the connectivity between the right amygdala and the occipital fusiform gyrus, paracingulate gyrus, and inferior frontal gyrus in adults from BL to 1 week PT (Fig. 4B; Supplementary Table S3). Exploratory whole-brain analyses revealed that MPH increased reactivity to the emotional face-matching task in the superior frontal gyrus and paracingulate cortex in children in the period of 8 weeks during treatment (DT) to 1 week after treatment. Additionally, a decrease in reactivity in the lateral occipital cortex was found in placebo-treated adults from BL to 1 week PT (Fig. 4C).

## Discussion

In this 4-month RCT in stimulant treatment-naive boys and men with ADHD, MPH did not influence internalizing symptoms or neural substrates underlying emotional processing, although MPH positively affected ADHD symptoms in both the children and adults, compared to placebo. Furthermore, we did not find that age modulated any of these effects. However, PPI analyses showed an increase of connectivity between the right amygdala and frontal regions only in the MPH-treated adults. In our exploratory whole-brain reactivity analyses we found small increases in cortico-limbic circuits in the MPH-treated children and in MPH-treated adults, we showed decreasing effects in the lateral occipital cortex. Interestingly, higher BL depressive and anxiety symptoms in adults predicted larger ADHD symptom reductions in the MPH but not the placebo condition. These results were independent of BL ADHD severity, providing further evidence for the important role of internalizing symptoms in obtaining clinical response and the role of ADHD medication herein.

Previous studies, including our research in the same sample of ADHD participants (BL only), showed that acute MPH administration normalized the heightened amygdala reactivity during emotional processing in individuals with ADHD (Posner *et al*., 2011a; Bottelier *et al*., 2017). In contrast, in the current study on prolonged MPH treatment, we did not find evidence for altered amygdala reactivity, nor was this response age-dependent. Previous studies comparing individuals with and without ADHD using emotional processing tasks have found mixed results, with some studies reporting increased left amygdala reactivity (Brotman *et al*., 2010; Posner *et al*., 2011b), whereas others only found significant results for either adults or children, or only in participants with certain comorbidities (Shaw *et al*., 2014). Consequently, functional impairments of the amygdala, and therefore also the influence of MPH thereon, may be heterogeneous and highly dependent on the task. Furthermore, studies have suggested that ADHD-related deficits in the PFC may be responsible for the deficient integration of information of regions responsible for perception and emotion recognition (Winston *et al*., 2003). Therefore, medication effects should be assessed within the functional network associated with the task.

Indeed, in our exploratory whole-brain connectivity analyses we found small yet specific effects in the MPH-treated children within cortico-limbic circuits. Although these findings require replication in larger samples, they indicate that MPH induces changes in top-down control processes, as MPH has been found before to primarily affect fronto-parietal circuits (Faraone *et al*., 2019). In line with our results, the “dyscontrol hypothesis” postulates that externalizing symptoms in ADHD are not emerging from direct dysfunctional emotional processing itself, but rather from executive dysfunction, affecting top-down processes, such as the capacity to suppress responses evoked by emotional stimuli (Posner *et al*., 2014). Research on internalizing symptoms and their neural correlates in ADHD participants is scarce. One study found that increased connectivity of the amygdala with prefrontal regions is associated with higher internalizing emotional regulation problems in children with ADHD (Uchida *et al*., 2015). Although we did not find changes in amygdala-prefrontal connectivity in children, in MPH-treated adults, we showed a significant increase in connectivity between the right amygdala and frontal regions, including paracingulate gyrus and inferior frontal gyrus. Additionally, this group showed increased connectivity between the right amygdala and the fusiform gyrus during the task. This pathway is thought to be important for emotional feedback from the amygdala during visual processing in the fusiform gyrus (Vuilleumier *et al*., 2004). While several studies have linked deficits in connectivity in this particular pathway to problems with emotional processing in various disorders (Herrington *et al*., 2011), future studies should consider investigating the influence of MPH on internalizing symptoms and its relation to the neural mechanisms of emotional processing in ADHD further as research on this topic is still scarce.

Children across both treatment conditions scored lower on scales of anxiety and depression during and after the trial. This finding points towards a general trial effect (Arkes and Harkness, 1980), including the consequences of a diagnosis and the subsequent support, rather than medication-specific improvements in these symptoms. It is important to note that the BL anxiety scores in this sample were in the clinical range for anxiety symptoms for most children (54% SCARED > 25), but their BL depression scores were identified as "none to mild" for 84% of the sample. However, in adults, the BL depression and anxiety scores were in the subclinical range for most (BDI: 83% less than mild depressive symptoms; BAI: 70% less than mild anxiety symptoms). Therefore, it is perhaps not surprising that anxiety symptoms did not change over the course of the trial.

Depressive symptoms in adults transiently decreased during the trial in the placebo condition. This effect was minimal and is likely due to the significant variance and individual differences in this measure. Despite the low prevalence of anxiety and depressive symptomatology, our results are of clinical importance. Contrary to previous preclinical and some human studies (Bolaños *et al*., 2003; Somkuwar *et al*., 2016), we did not observe an increase in depressive and anxiety symptoms in the MPH condition during the 16 weeks in this well-controlled trial. This is in line with another long-term (3-year) study, which found a reduced risk for developing depressive symptoms associated with previous medication (Chang *et al*., 2016). Moreover, the effect of MPH on internalizing symptoms is likely patient-specific and Coughlin *et al*. (2015) argued in their meta-analysis that the positive impact on anxiety symptoms outweighs the risk of psychostimulants inducing anxiety in children with ADHD, and so far, the causal link between stimulant treatment and internalizing symptoms thus remains debated.

Interestingly, higher depression and anxiety symptoms at BL predicted a larger ADHD symptom-severity reduction during the trial (week 8) and after the trial end (1 week PT) in the adult MPH condition, but not in the adult placebo group or in children. The current guidelines for treating adult ADHD state that comorbid depression and anxiety require treatment before starting stimulant medication, as stimulants could introduce internalizing symptoms as a side-effect (Kooij *et al*., 2004; Kollins, 2009). In contrast, we did not find an MPH-induced increase in internalizing symptoms in adults or children with ADHD, and therefore do not confirm previous findings (Molina *et al*., 2009; Fredriksen, 2014). As such, we could argue that worries about MPH introducing internalizing symptoms in children or adults might be unwarranted and MPH should be considered as a potential treatment for adults with ADHD and anxiety and/or depression comorbidities. However, close monitoring of side-effects should always be ensured and future studies in samples with more severe internalizing symptoms should replicate these findings. Notably, adults' depression and anxiety scores at BL in our study were in the subclinical range. Therefore, future studies should additionally consider investigating the interactions between MPH, depression and anxiety, and ADHD symptoms within an ADHD population with more severe internalizing symptoms.

These results are conflicting with the recent findings of Masi *et al*., who reported that higher emotional dysregulation at BL, as assessed by the CBCL dysregulation profile (including symptoms of anxiety/depression, aggression, and inattention), predicted higher ADHD symptoms at follow-up after 4 weeks of MPH treatment in children and adolescents (Masi *et al*., 2020). In their trial, individuals were followed for 4 weeks of MPH treatment, whereas we assessed our participants (both children and adults) after 8 and 17 weeks. Additionally, the operationalization of emotion regulation problems differed between the two studies; while Masi *et al*. assessed emotion dysregulation defined as a combination of internalizing and externalizing symptoms (depression, anxiety, attention, and aggression) and considered absolute values of ADHD symptom severity at PT, we focused on internalizing symptoms of anxiety and depression in relation to changes in ADHD symptom severity, possibly explaining the differences in results.

A critical strength of our current study is its design. To rule out the influence of a history of medication use, we included only stimulant treatment-naive individuals. For ethical reasons, we could not extend the follow-up period to more than 4 months and did not include healthy control participants in our study; therefore, we cannot argue how amygdala reactivity changed compared to healthy control participants. Further limitations of our study are that its results cannot be extrapolated to all children and adults with ADHD, as we only studied male participants within a specific age range. We chose to include only male participants to limit participant variation. Females and males differ considerably in their patterns of brain growth (Giedd *et al*., 1999) and ADHD is most prevalent in male individuals (Polancyk *et al*., 2007). Additionally, the fact that patients in the MPH groups were prescribed short-acting MPH, and that DT scans were carried out throughout the day, may have resulted in increased variability in fMRI activity within these groups at that timepoint. However, this is not reflected in differences in variance between the groups. Even though we applied advanced and state-of-the-art motion correction methods, we had to exclude several scans due to motion in the MRI scanner; consequently, framewise displacement did not differ between groups included in the analysis (Supplementary Table S1). Furthermore, internalizing symptoms are known to change over development, and as such, its operationalization varies in children and adults, even though we assume similar underlying neural processes (Shaw *et al*., 2014). This makes a comparison of the symptomatology between the age groups challenging, especially keeping in mind the complexity of the multiple domains that might span emotion regulation problems (Graziano and Garcia, 2015). Future studies should clarify the effects of prolonged stimulant therapy on the differential effects on internalizing and externalizing comorbidities in ADHD, both separately and together, and their underlying neural mechanisms.

In conclusion, we did not find evidence for the effects of prolonged MPH on internalizing symptoms nor neural substrates underlying emotional processing. Nevertheless, we did demonstrate that MPH improved ADHD symptoms the most in adults with the highest depressive and anxiety symptoms at BL, suggesting that adult ADHD patients with comorbidities could also benefit from treatment with MPH. Furthermore, we did not confirm that MPH treatment increased internalizing symptoms in either children or adults, suggesting that worries about (early) prescription of MPH might be unwarranted. Nevertheless, future studies in an ADHD population with more severe internalizing symptoms should confirm these findings to guide treatment in patients at risk for, or presenting with, comorbidities.

## Supplementary Material

kkab013_Supplemental_File
