# Supplementary material for: Effects of prolonged methylphenidate treatment on amygdala reactivity and connectivity: a randomized controlled trial in stimulant treatment-naive, male participants with ADHD
Source: Psychoradiology. 2021 Oct 22;1(3):152–63. doi: 10.1093/psyrad/kkab013 (PMC10917223; doi:10.1093/psyrad/kkab013)
Supplement: kkab013_Supplemental_File [file kkab013_Supplemental_File.docx]

# Supplementary methods

## Participants

Boys aged 10-12 years and men aged 23-40 years were included. Inclusion criteria were meeting criteria for a diagnosis of and requiring treatment with medication for ADHD (Inattentive, Hyperactive/Impulsive or Combined subtype). The diagnosis was determined by an experienced clinician based on the Diagnostic and Statistical Manual of Mental Disorders (DSM-IV; (American Psychiatric Association 1994)), which was confirmed with a (semi-)structured interview (Ferdinand and van der Ende 1998) in children; Diagnostic Interview for Adult ADHD (DIVA; (Kooij 2012)). The DSM-IV requirement of at least six inattention or hyperactivity/impulsivity symptoms was applied to both children and adults. Participants were not eligible when they had received clinical treatment influencing the DA system (for adults before age 23), such as stimulants, neuroleptics, antipsychotics, D2/D3 agonists, or when they had a current or previous dependency on drugs that influence the DA system (for adults before age 23). Other exclusion criteria were an estimated IQ < 80 (Block Design and Vocabulary subtests of the WISC-III-R (Kort et al. 2002), Dutch Adult Reading Test (Schmand et al. 1992)., and/or a history of significant medical or neurological trauma or illness (see Figure 2 for a CONSORT flow diagram).

## fMRI paradigm

Subjects performed an emotion recognition fMRI paradigm at three different time points during the trial (Figure 1). To further minimize learning effects, a practice run was presented before the first MRI scan. Two versions of the tasks were used to overcome learning effects.

The emotion recognition paradigm consisted of a blocked design and was adapted from a task previously used to assess drug effects on amygdala reactivity (Hariri et al. 2002; Bottelier et al. 2017). The emotional stimuli consisted of angry and fearful faces, whereas the neutral stimuli consisted of ellipses assembled from scrambled faces. Two blocks of emotional stimuli were interleaved with three neutral blocks, each block (30s) containing six trials (5s) (6 trials per block x 5 blocks = 30 trials (15 min.)). For each emotional trial, three stimuli were presented simultaneously, and subjects had to decide which one of the lower two stimuli expressed the same emotion as the target stimuli presented above. Similarly, for each neutral trial, three stimuli were presented, but subjects had to decide which of the bottom two ellipses was identically oriented to the target ellipse. During the task, reaction time to button press and accuracy were recorded.

**Supplementary Figure 1.| fMRI task paradigm.**
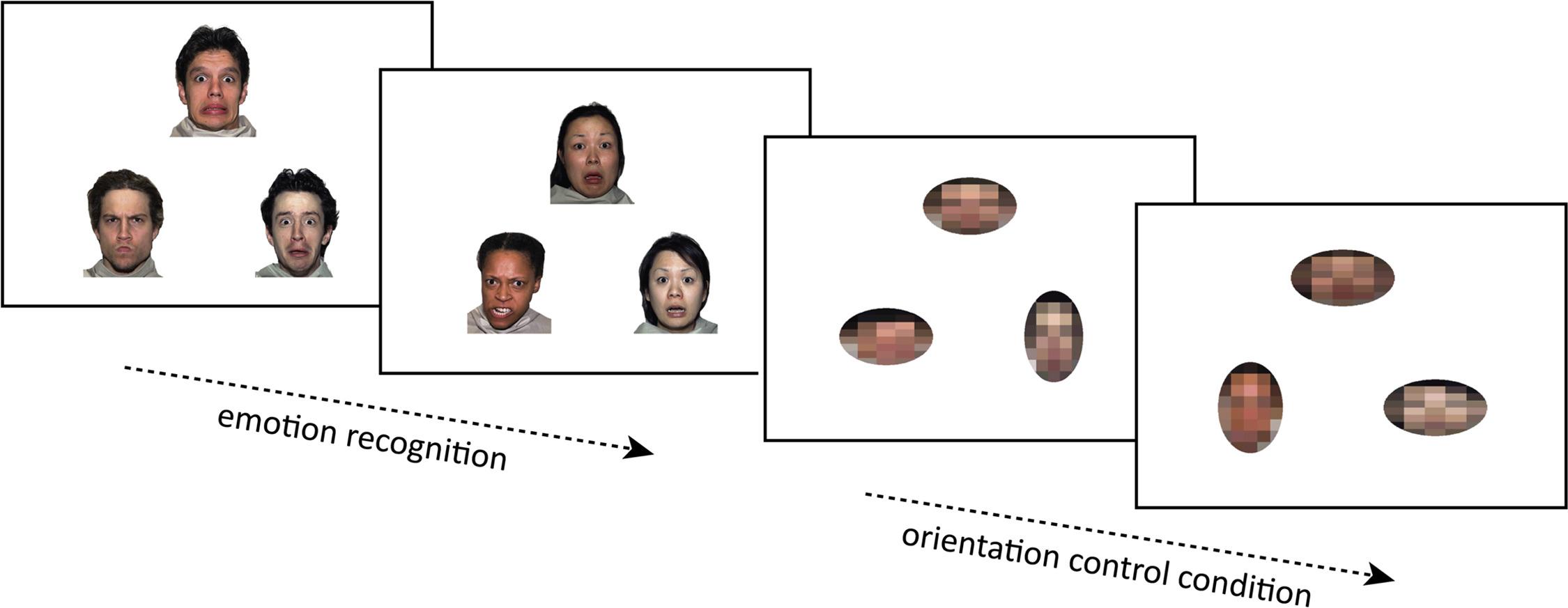


## MRI acquisition

The MRI study was performed on a 3·0 T Philips scanner (Philips Healthcare, Best, The Netherlands) using an 8-channel receive-only head coil. A high-resolution 3D T1-weighted anatomical scan was acquired for registration purposes, and fMRI data were obtained using a single-shot echo-planar imaging sequence Parameters were: TR/TE=2300/30 ms, resolution=2·3×2.3×3 mm, 39 sequential slices, FOV=220x220x117 mm, GE-EPI read-out, no gap, 80° flip angle, 70 dynamics were used.

## MRI preprocessing

Preprocessing was performed using FMRIPREP v1.2.3. Each T1w scan was bias-corrected, skull-stripped, and subsequently normalized to MNI space using non-linear registration. Functional data preprocessing included motion correction using FLIRT and distortion correction using an implementation of the TOPUP technique using 3dQwarp. This was followed by co-registration to the corresponding T1w using boundary-based registration with 9 degrees of freedom. Motion correcting transformations, field distortion correcting warp, BOLD-to-T1w transformation, and T1w-to-template (MNI) warp were concatenated and applied in a single step using antsApplyTransforms (ANTs v2.1.0) with Lanczos interpolation. Independent component analysis (ICA) based on Automatic Removal Of Motion Artifacts (AROMA) was used to generate data that was non-aggressively denoised. Subsequently, data were spatially smoothed (6mm FWHM), and a high pass-filter (100s) was applied using FSL.

First-level analyses were performed by modeling the signal changes using the stimulation paradigm (faces versus shapes), convolved with a canonical hemodynamic response function. Data from subjects with extreme motion (framewise displacement > 1mm) were removed from the analysis.

**Supplementary Figure 2.| Treatment assignment.**


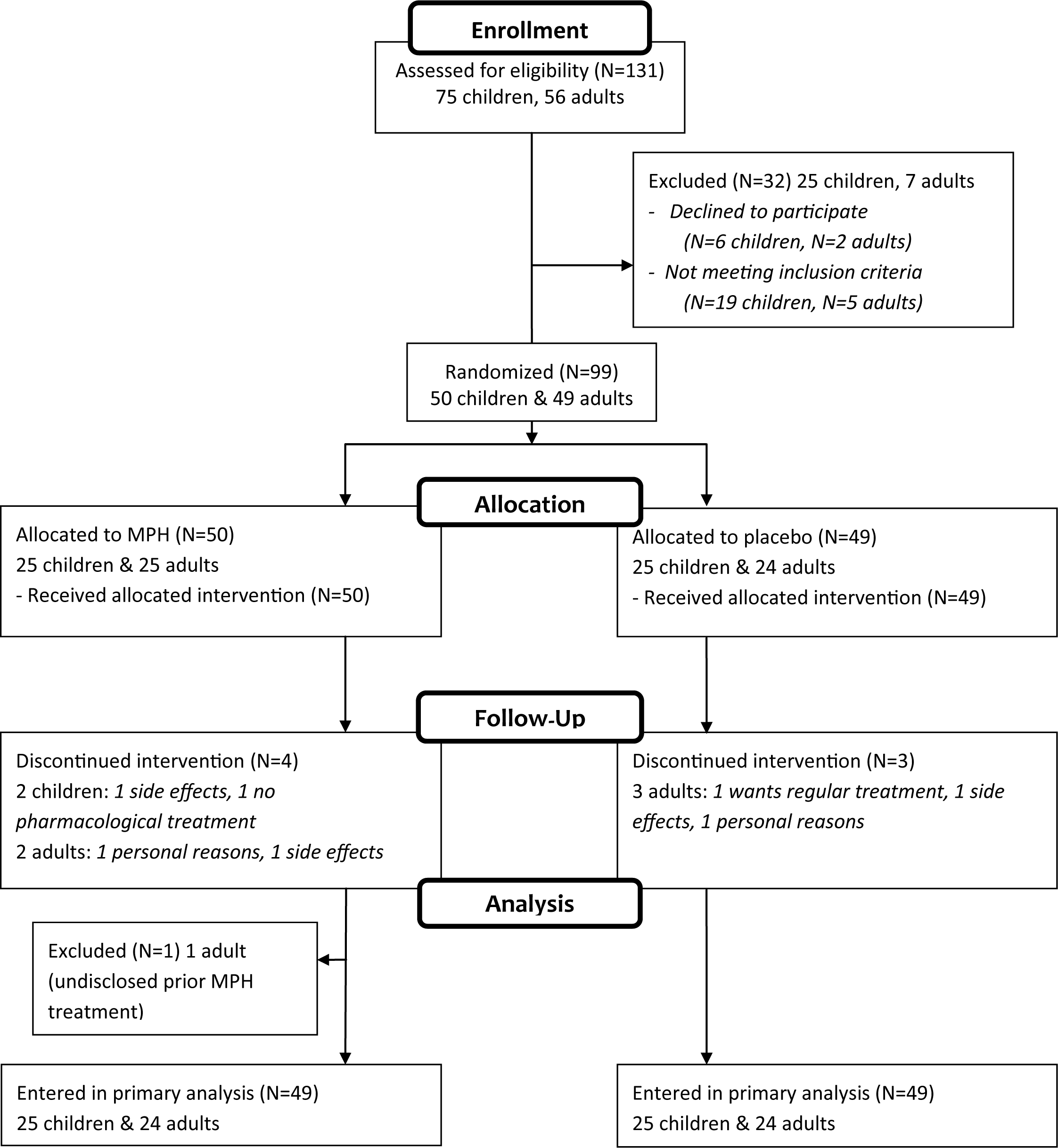


# Supplementary Results

**Supplementary Table 1.| Characteristics of the participants included in the fMRI Analysis of the randomized controlled trial at Baseline.**

|  | Children  MPH | placebo | Adults  MPH | placebo |
| --- | --- | --- | --- | --- |
|  | n=17 | n=15 | n=21 | n=22 |
| Age (y)  Estimated IQ^1^ | mean±SD  11.4±0.9  107.3±20.9 | mean±SD  11.3±1.1  101.9±13.8 | mean±SD  28.04±4.5  107.2±7.9 | mean±SD  28.8±5.1  107.5±6.4 |
| **ADHD subtype (N)**  Inattentive  Hyperactive/impulsive  Combined | 9  0  8 | 9  0  6 | 10  0  11 | 4  0  18 |
| **ADHD symptoms**  DBD-RS Inattention  DBD-RS Hyperactivity  ADHD-SR | 21.9±3.6  14.3±5.5  - | 23.5±2.6  15.1±7.4  - | -  -  33.9±10.1 | -  -  31.5±10.1 |
| Depressive symptoms²  Anxiety symptoms² | 7.8±4.8  27.1±19.4 | 9.7±5.3  31.4±18.83 | 6.5±5.5  9.9±6.8 | 7.7±6.1  9.0±8.2 |
| Adherence | 83%±19 | 79%±20 | 90%±8 | 86%±9 |
| **Framewise displacement (mm)**  Baseline  During Treatment  Post-Treatment | 0.37±0.24  0.23±0.08  0.34±0.29 | 0.46±0.24  0.35±0.20  0.39±0.28 | 0.15±0.10  0.10±0.05  0.16±0.12 | 0.12±0.05  0.15±0.10  0.17±0.09 |

^1^for children: Wechsler Intelligence Scale for Children (WISC) (Kort et al. 2002); for adults: National Adults Reading Test (NART) (Schmand et al. 1992); DBD-RS=disruptive behavior disorder rating scale (Pelham Jr. et al. 1992); ADHD-SR=Attention Deficit Hyperactivity Disorder-Self Report (Kooij 2012);

²Depressive symptoms and anxiety symptoms: children: Child Depression Inventory (CDI) (Kovacs 1985); Screen for Child Anxiety Related Disorders (SCARED) (Muris et al. 1998); adults: Beck's Depression Inventory (BDI) (Beck et al. 1961); Beck's Anxiety Inventory (BAI) (Beck et al. 1988)

**Supplementary Table 2.| Statistics.**

|  | session * MED (+FD) | Main effect session (BL/DT/PT) (+FD) | Main effect medication/placebo (+FD) |
| --- | --- | --- | --- |
| **Children**: |  |  |  |
| *left amygdala reactivity* | F(5,70.14)=1.23, *p=*0.30, ΔBIC=18.09 | F(2,63.61)=0.68, *p=*0.51, ΔBIC=9.49 | F(1,39.45)=0.14, *p=*0.70, ΔBIC=6.19 |
| *right amygdala reactivity* | F(5,68.46)=1.22, *p=*0.31, ΔBIC=16.45 | F(2,60.58)=1.27, *p=*0.29, ΔBIC=6.5 | F(1,40.54)=0.85, *p=*0.36, ΔBIC=3.65 |
| *Accuracy face trials* | F(5,72.66)=0.99, *p=*0.50, ΔBIC=18.19 | F(2,67.70)=1.00, *p=*0.22, ΔBIC=5.98 | F(1,36.40)=1.00, *p=*0.96, ΔBIC=4.59 |
| *Accuracy shape trials* | F(3,68.83)=1.24, *p=*0.30, ΔBIC=9.8 | F(2,67.01)=6.15, ***p<*0.01**, ΔBIC=-3.2 | F(1,37.12)=0.38, *p=*0.54, ΔBIC=4.2 |
| *Reaction time face trials* | F(5,66.93)=1.06, *p=*0.39, ΔBIC=17.45 | F(2,58.61)=2.51, *p=*0.09, ΔBIC=4.27 | F(1,40.83)=0.01, *p=*0.93, ΔBIC=4.56 |
| *Reaction time shape trials* | F(5,87.10)=0.84, *p=*0.52, ΔBIC=17.51 | F(2,77.09)=1.78, *p=*0.16, ΔBIC=5.31 | F(1,43.89)=0.02, *p=*0.89, ΔBIC=4.36 |
| *CDI* | F(3,96.13)=0.63, *p=*0.60, ΔBIC=11.63 | F(2,90.77)=38.17, ***p<*0.01**, ΔBIC=-4.69 | F(1,46.88)=0.09, *p=*0.77, ΔBIC=49.91 |
| *SCARED* | F(3,97.39)=0.51, *p=*0.67, ΔBIC=12.49 | F(2,92.72)=22.70, ***p<*0.01**, ΔBIC=-29.09 | F(1,46.99)=0.03, *p=*0.87, ΔBIC=5.10 |
| *DBD-RS Inattention* | F(2,83.49)=5.47, ***p<*0.01**, ΔBIC=-69.10 | F(2,85.25)=61.44, ***p<*0.01**, ΔBIC=-38.4 | F(1,44.08)=12.69, ***p<*0.01**, ΔBIC=-4.1 |
| *DBD-RS Hyperactivity* | F(3,90.33)=2.48, *p=*0.07, ΔBIC=7.26 | F(2,83.87)=30.80, ***p<*0.01**, ΔBIC=-32.28 | F(1,45.51)=3.09, *p=*0.09, ΔBIC=2.4 |
| **Adults:** |  |  |  |
| *left amygdala reactivity* | F(5,92.69)=0.69, *p=*0.63, ΔBIC=20.54 | F(2,83.85)=0.86, *p=*0.43, ΔBIC=8.21 | F(1,42.50)=1.12, *p=*0.30 ΔBIC=3.78 |
| *right amygdala reactivity* | F(5,92.08)=1.17, *p=*0.33, ΔBIC=18.17 | F(2,82.64)=0.85, *p=*0.43, ΔBIC=8.26 | F(1,43.12)=2.57, *p=*0.12, ΔBIC=2.21 |
| *Accuracy face trials* | F(5,85.32)=0.09, *p=*0.66, ΔBIC=20.33 | F(2,76.39)=0.09, *p=*0.66, ΔBIC=8.63 | F(1,43.15)=1.15, *p=*0.19, ΔBIC=2.93 |
| *Accuracy shape trials* | F(3,82.97)=0.04, *p=*0.99, ΔBIC=17.06 | F(2,81.06)=3.33**,** *p=*0.04, ΔBIC=2.88 | F(1,41.92)=0.08, *p=*0.78, ΔBIC=4.70 |
| *Reaction time face trials* | F(3,81.50)=1.36, *p=*0.26, ΔBIC=10.08 | F(2,72.83)=5.97, ***p<*0.01**, ΔBIC=-2.07 | F(1,44.81)=0.01, *p=*0.97, ΔBIC=5.02 |
| *Reaction time shapes trials* | F(5,67.13)=1.02, *p=*0.42, ΔBIC=19.52 | F(2,59.09)=1.87, *p=*0.17, ΔBIC=5.96 | F(1,43.65)=0.19, *p=*0.65, ΔBIC=4.75 |
| *BAI* | F(5,96.99)=1.02, *p=*0.41, ΔBIC=19.57 | F(2,84.82)=2.01, *p=*0.14, ΔBIC=6.20 | F(1,46.82)=0.06, *p=*0.81, ΔBIC=4.76 |
| *BDI* | F(3,88.57)=1.18, *p=*0.32, ΔBIC=12.75 | F(2,43.61)=4.05, ***p=*0.02**, ΔBIC=1.84 | F(1,43.61)=1.04, *p=*0.31, ΔBIC=3.79 |
| *ADHD-SR* | F(2,78.3)=4.82, ***p=*0.01**, ΔBIC=-36.59 | F(2,80.48)=31.46, ***p<*0.01**, ΔBIC=-38.97 | F(1,43.95)=3.18, *p=*0.08, ΔBIC=2.38 |

**Supplementary Figure 3.| Mean and confidence interval of the dose of MPH treatment or placebo over the whole trial, including data points per participant.**

**
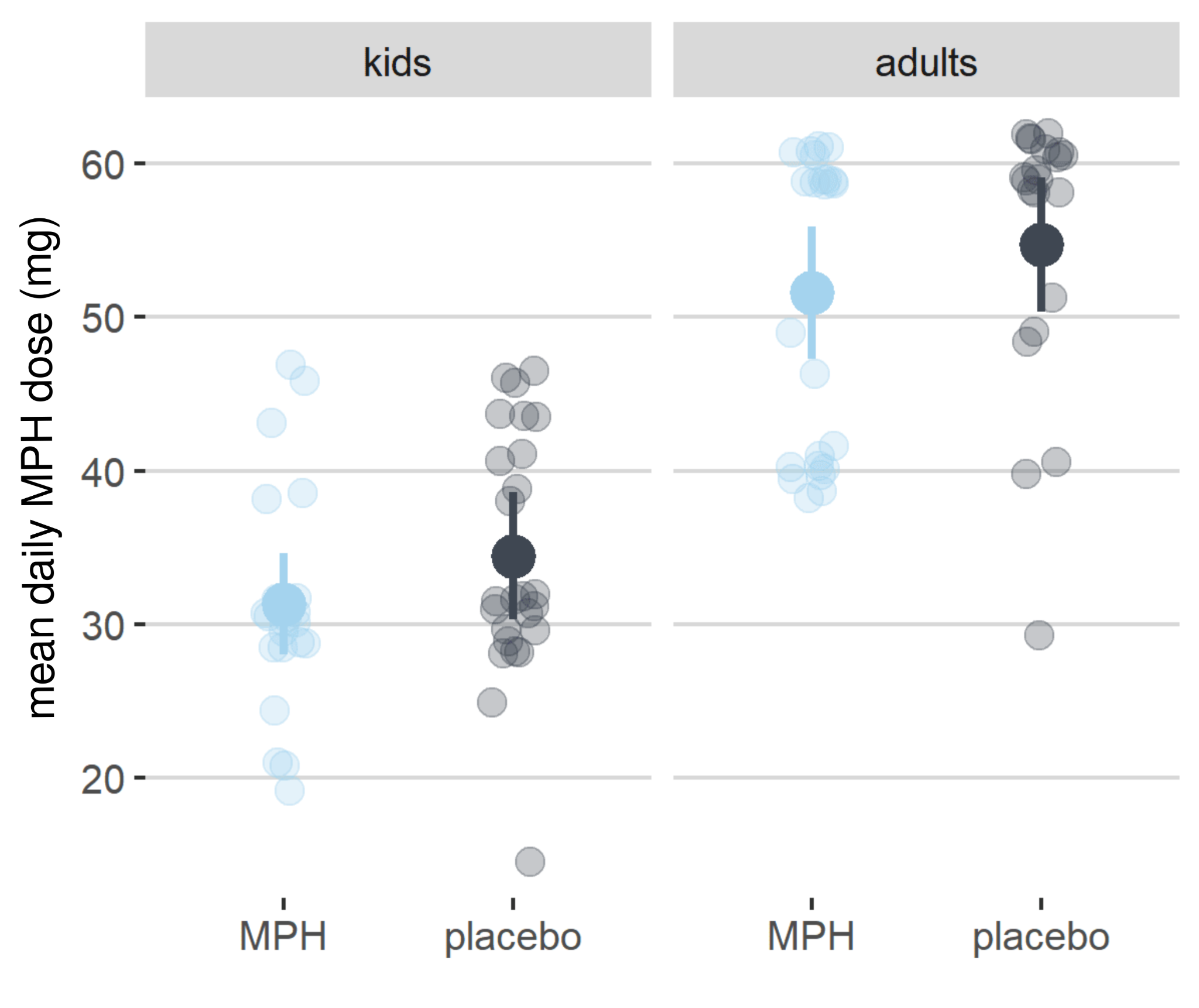
**

**Supplementary Figure 4 | Accuracy and reaction time measures from the fMRI task.**


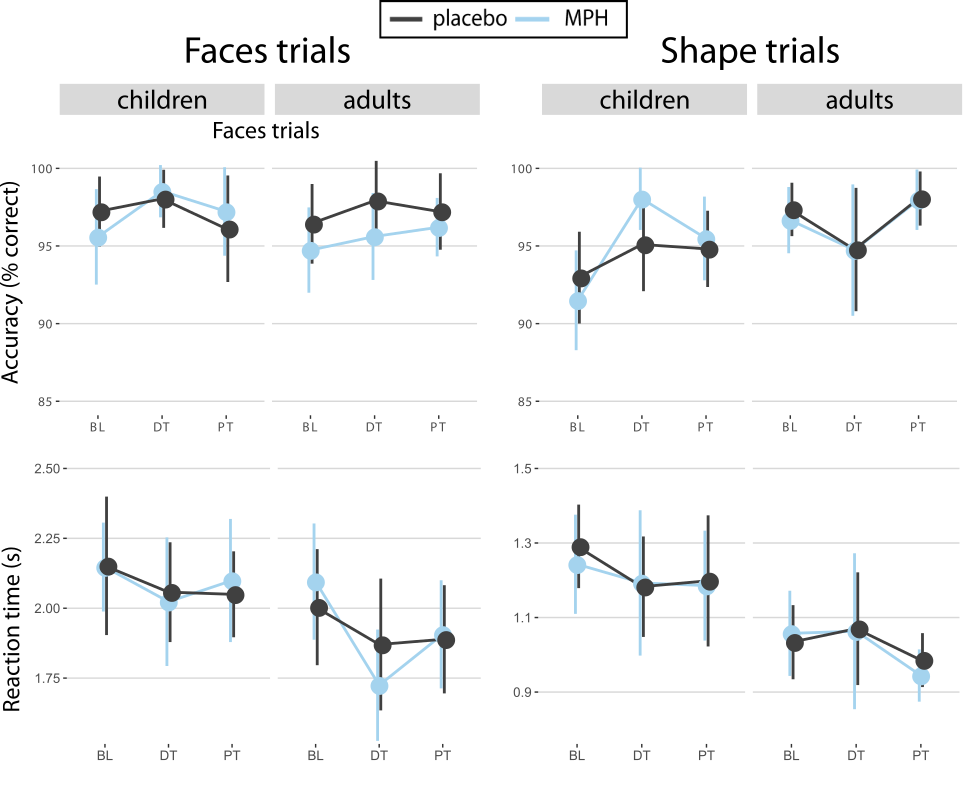


**Supplementary Table 3.| PPI whole-brain analysis and exploratory task reactivity whole-brain analysis of the emotion recognition taks (Faces > shapes).**

| *Seed Cluster-index # of voxels Max. T-value MNI-coordinates Brain areas*  *X Y Z* | | | | | | | | | |
| --- | --- | --- | --- | --- | --- | --- | --- | --- | --- |
| ***Connectivity*** |  |  |  |  |  |  |  |  |  |
| ***PT >BL*** |  |  |  |  |  |  |  |  |  |
| *Adults MPH* | Right amygdala | 1 | 38 | 4.27 | -8 | 46 | 16 | 67% Paracingulate Gyrus,  13% Cingulate Gyrus, anterior division,  1% Superior Frontal Gyrus |  |
|  |  | 2 | 45 | 4.27 | -42 | 18 | 32 | 45% Middle Frontal Gyrus,  7% Inferior Frontal Gyrus, pars opercularis,  2% Inferior Frontal Gyrus, pars triangularis |  |
|  |  | 3 | 196 | 5.45 | -26 | -78 | -16 | 69% Occipital Fusiform Gyrus,  5% Lingual Gyrus,  4% Lateral Occipital Cortex, inferior division |  |
| ***Reactivity*** |  |  |  |  |  |  |  |  |  |
| ***PT> BL*** |  |  |  |  |  |  |  |  |  |
| *Adults placebo* | Whole-brain | 1 | 23 | 4.68 | 52 | -74 | 22 | 55% Lateral Occipital Cortex, superior division,  2% Lateral Occipital Cortex, inferior division |  |
|  |  | 2 | 42 | 5.38 | 30 | -88 | 30 | 33% Lateral Occipital Cortex, superior division,  25% Occipital Pole |  |
| ***DT > PT*** |  |  |  |  |  |  |  |  |  |
| *MPH kids* | Whole-brain | 1 | 40 | 4.28 | 2 | 22 | 50 | 29% Superior Frontal Gyrus,  22% Paracingulate Gyrus |  |
|  |  | 2 | 122 | 4.97 | 0 | 36 | 28 | 66% Paracingulate Gyrus,  18% Cingulate Gyrus, anterior division |  |

**Supplementary Table 4.| Correlations of amygdala reactivity and clinical measures per session (significance level alpha=0.008 after bonferroni correction per age group and session (number of tests=6)).**

|  | ***BL***  *Placebo* | *MPH* | ***DT***  *Placebo* | *MPH* | ***PT***  *Placebo* | *MPH* |
| --- | --- | --- | --- | --- | --- | --- |
| ***Left amygdala*** |  |  |  |  |  |  |
| ***Children*** |  |  |  |  |  |  |
| *Depressive symptoms* | t(12)=-0.41, *p=*0.69 | t(15)=-0.66, *p=*0.52 | t(16)=0.20, *p=*0.84 | t(15)=-0.32, *p=*0.75 | t(13)=0.09, *p=*0.93 | t(14)=-1.13, *p=*0.28 |
| *Anxiety symptoms* | t(13)=-1.23, *p=*0.24 | t(15)=-0.24, *p=*0.81 | t(16)=0.66, *p=*0.52 | t(13)=-0.13, *p=*0.90 | t(14)=-0.90, *p=*0.39 | t(14)=-0.15 ,*p=*0.89 |
| *ADHD: inattention subscale* | t(13)=1.38, *p*=0.19 | t(14)=0.04, *p*=0.97 | t(13)=0.31, *p*=0.17 | t(13)=-0.79, *p*=0.44 | t(11)=0.53, *p*=0.61 | t(13)=0.36, *p*=0.72 |
| *ADHD: hyperactivity subscale* | t(13)=1.42, *p*=0.18 | t(14)=0.15, *p*=0.88 | t(13)=0.31, *p*=0.76 | t(13)=-0.56, *p*=0.58 | t(11)=0.27, *p*=0.79 | t(13)=-0.75, *p*=0.46 |
| ***Adults*** |  |  |  |  |  |  |
| *Depressive symptoms* | t(19)=0.96, *p=*0.35 | t(19)=1.01, *p=*0.33 | t(18)=-0.31, *p=*0.76 | t(18)=0.71, *p=*0.49 | t(18)=1.26, *p=*0.22 | t(19)=0.69, *p=*0.49 |
| *Anxiety symptoms* | t(20)=2.62, *p=*0.02 | t(18)=0.62, *p=*0.54 | t(16)=0.24, *p=*0.81 | t(18)=-0.29, *p=*0.78 | t(17)=0.23, *p=*0.82 | t(19)=0.60, *p=*0.55 |
| *ADHD symptoms* | t(19)=1.45, *p*=0.16 | t(16)=1.47, *p*=0.16 | t(15)=1.46, *p*=0.17 | t(17)=-0.28, *p*=0.79 | t(18)=-0.42, *p*=0.68 | t(18)=0.37, *p*=0.72 |
| ***Right amygdala*** |  |  |  |  |  |  |
| ***Children*** |  |  |  |  |  |  |
| *Depressive symptoms* | t(12)=0.03, *p=*0.98 | t(15)=0.20, *p=*0.84 | t(16)=-0.17, *p=*0.87 | t(15)=0.57, *p=*0.58 | t(13)=0.29, *p=*0.78 | t(14)=0.68, *p=*0.50 |
| *Anxiety symptoms* | t(13)=-0.68, *p=*0.51 | t(15)=-0.40, *p=*0.70 | t(16)=0.97, *p=*0.35 | t(15)=0.57, *p=*0.58 | t(13)=1.13, *p=*0.28 | t(14)=-0.65, *p=*0.52 |
| *ADHD: inattention subscale* | t(13)=1.05, p=0.31 | t(14)=-1.06, *p*=0.31 | t(13)=0.06, *p*=0.95 | t(13)=1.21, *p*=0.74 | t(11)=-0.44, *p*=0.67 | t(13)=0.15, *p*=0.88 |
| *ADHD: hyperactivity subscale* | t(13)=1.42, *p*=0.18 | t(14)=-0.95, *p*=0.36 | t(13)=-1.48, *p*=0.16 | t(13)=0.34, *p*=0.25 | t(11)=-1.29, *p*=0.22 | t(13)=-0.62, *p*=0.55 |
| ***Adults*** |  |  |  |  |  |  |
| *Depressive symptoms* | t(19)=0.73, *p=*0.47 | t(19)=0.69, *p=*0.50 | t(18)=-1.19, *p=*0.25 | t(18)=0.56, *p=*0.58 | t(18)=0.55, *p=*0.59 | t(19)=-0.45, *p=*0.66 |
| *Anxiety symptoms* | t(20)=0.88, *p=*0.39 | t(18)=0.73, *p=*0.47 | t(16)=-0.22, *p=*0.83 | t(18)=0.61, *p=*0.55 | t(17)=-0.41, *p=*0.69 | t(19)=-0.52, *p=*0.61 |
| *ADHD symptoms* | t(19)=0.92, *p*=0.37 | t(16)=1.36, *p*=0.19 | t(15)=1.48, *p*=0.16 | t(17)=-0.16, *p*=0.88 | t(18)=-1.06, *p*=0.30 | t(18)=-2.12, *p*=0.05 |

# References

American Psychiatric Association (1994) Diagnostic and Statistical Manual of Mental Disorders (4th edn, DSM-IV). Washington, DC Am Psychiatr Assoc

Beck AT, Epstein N, Brown G, Steer RA (1988) An inventory for measuring clinical anxiety: psychometric properties. J Consult Clin Psychol 56:893–897

Beck AT, Ward CH, Mendelson M, et al (1961) An inventory for measuring depression. Arch Gen Psychiatry 4:561–71

Bottelier MA, Schrantee A, Ferguson B, et al (2017) Age-dependent effects of acute methylphenidate on amygdala reactivity in stimulant treatment-naive patients with Attention Deficit/Hyperactivity Disorder. Psychiatry Res - Neuroimaging 269:36–42. https://doi.org/10.1016/j.pscychresns.2017.09.009

Ferdinand R, van der Ende J (1998) DISC-IV Diagnostic Interview Schedule for Children [Dutch translation NIMH-DISC-IV]. Afdeling Kinder- en Jeugdpsychiatrie, Sophia Kinderziekenhuis/Academisch Ziekenhuis Rotterdam, Rotterdam, the Netherlands

Hariri AR, Tessitore A, Mattay VS, et al (2002) The amygdala response to emotional stimuli: a comparison of faces and scenes. Neuroimage 17:317–23

Kooij J (2012) Adult ADHD: Diagnostic assessment and treatment. Springer-Verlag, London

Kort W, Compaan EL, Bleichrodt N, et al (2002) WISC-III NL. Handleiding. London Psychol Corp

Kovacs M (1985) The Children’s Depression Inventory (CDI). Psychopharmacol Bull 21:995–8

Muris P, Merckelbach H, Van Brakel A, et al (1998) The screen for child anxiety related emotional disorders (SCARED): Relationship with anxiety and depression in normal children. Pers Individ Dif 24:451–456. https://doi.org/10.1016/S0191-8869(97)00217-1

Pelham Jr. WE, Gnagy EM, Greenslade KE, Milich R (1992) Teacher ratings of DSM-III-R symptoms for the disruptive behavior disorders. J Am Acad Child Adolesc Psychiatry 31:210–218

Schmand B, Lindeboom J, van Harskamp F (1992) Dutch Adult Reading Test. Lisse Swets en Zeitlinger
